# Supplementary material for: riboCIRC: a comprehensive database of translatable circRNAs
Source: Genome Biol. 2021 Mar 8;22:79. doi: 10.1186/s13059-021-02300-7 (PMC7938571; doi:10.1186/s13059-021-02300-7)
Supplement: Supplementary file 1 — Additional file 1: Table S1. Summary of Ribo-seq and matched RNA-Seq datasets used in this study. [file 13059_2021_2300_MOESM1_ESM.doc]

**Table S1. Summary of Ribo-seq and matched RNA-Seq data sets used in this study.**

| **Species** | **Number of studies** | **Number of RNA-seq samples** | **Number of Ribo-seq samples** |
| --- | --- | --- | --- |
| Human | 84 | 621 | 636 |
|  | GSE21992,GSE69047,GSE69602,GSE69906,GSE59820,GSE63591,GSE70211,GSE62247,GSE35469,GSE73565,GSE77292,GSE77315,GSE77317,GSE77347,GSE78959,GSE78960,GSE79664,GSE79804,GSE81802,GSE82232,GSE83493,GSE41605,GSE42509,GSE87328,SRA492656,GSE94454,GSE45785,GSE96643,GSE96714,GSE96716,GSE97140,GSE97384,GSE46613,GSE100007,GSE101760,GSE102040,GSE48785,GSE48933,GSE49339,SRA096542,GSE49716,SRA099816,GSE51584,GSE52447,GSE52809,GSE55195,GSE56148,GSE56887,GSE56924,GSE59817,GSE59818,GSE59819,GSE60426,GSE51424,GSE61375,GSE63570,GSE64962,GSE65778,GSE65885,GSE65912,GSE66809,GSE67902,GSE133111,GSE112705,GSE121391,GSE125086,GSE129869,GSE112085,GSE125114,GSE127713,GSE121952,GSE113695,GSE123564,GSE112305,GSE115647,GSE123539,GSE122071,GSE112295,GSE113171,GSE118239,GSE111866,GSE133925,GSE106483,GSE119615 | | |
| Mouse | 57 | 551 | 565 |
|  | PRJEB12126,PRJEB17636,PRJEB7207,PRJEB7276,GSE22001,GSE30839,GSE68265,GSE69699,GSE69800,GSE71333,GSE72064,GSE72066,GSE36892,GSE37111,GSE74537,GSE74683,GSE60930,GSE80156,GSE81283,GSE83332,GSE83351,GSE84112,GSE41246,GSE41785,GSE50983,GSE89011,GSE89108,GSE89184,GSE94385,GSE99787,GSE102659,GSE102890,GSE51424,GSE52809,GSE53743,GSE58423,GSE60426,GSE67305,GSE112185,GSE108331,GSE116221,GSE114064,GSE110618,GSE105147,GSE112223,GSE97286,GSE123919,GSE120762,GSE112502,GSE120097,GSE119365,GSE112766,GSE116233,GSE125725,GSE110866,GSE115526,GSE119567 | | |
| *Saccharomyces cerevisiae* | 39 | 384 | 391 |
|  | GSE13750,GSE69414,GSE70259,GSE34082,GSE34438,GSE74393,GSE76117,GSE61753,GSE81269,GSE81932,GSE81966,GSE84746,GSE85036,GSE85198,GSE85944,GSE87614,GSE87892,GSE91068,GSE100626,GSE52119,GSE50049,GSE108334,GSE51532,GSE52968,GSE53313,GSE55400,GSE56622,PRJNA245106,PRJNA254353,GSE63789,GSE66411,GSE67387,GSE125038,GSE114892,GSE109734,GSE122039,GSE115366,GSE102837,GSE104506 | | |
| *Escherichia coli* | 13 | 78 | 81 |
|  | PRJDB2960,PRJEB7301,GSE68762,GSE72899,GSE77617,GSE85540,GSE88725,GSE90056,GSE53767,GSE56372,GSE51052,GSE58637,GSE119454 | | |
| *Arabidopsis* | 7 | 27 | 27 |
|  | GSE69802,GSE81332,GSE86581,GSE43703,GSE98610,GSE50597,GSE109122 | | |
| *Schizosaccharomyces pombe* | 5 | 27 | 27 |
|  | PRJEB21099,PRJEB5150,PRJEB5263,GSE98934,GSE52809 | | |
| *Caenorhabditis elegans* | 4 | 35 | 35 |
|  | SRA049309,PRJNA170771,GSE48140,GSE67387 | | |
| Drosophila | 4 | 31 | 31 |
|  | GSE83616,GSE99920,GSE49197,GSE52799 | | |
| Rat | 4 | 43 | 43 |
|  | PRJEB7498,GSE60752,GSE66715,GSE129924 | | |
| *Trypanosoma brucei* | 3 | 20 | 22 |
|  | PRJEB4801,GSE72463,GSE57336 | | |
| Zebrafish | 3 | 30 | 33 |
|  | GSE34743,GSE52809,GSE53693 | | |
| *Caulobacter crescentus* | 2 | 8 | 8 |
|  | GSE68200,GSE54883 | | |
| *Staphylococcus aureus* | 2 | 12 | 16 |
|  | GSE74197,GSE57175 | | |
| *Salmonella enterica* | 2 | 6 | 6 |
|  | GSE87871,GSE91066 | | |
| *Crytococcus neoformans* | 1 | 6 | 6 |
|  | GSE133125 | | |
| *Halobacterium salinarum* | 1 | 12 | 12 |
|  | PRJNA413990 | | |
| Chinese hamster | 1 | 6 | 6 |
|  | GSE79512 | | |
| *Pseudomonas aeruginosa* | 1 | 12 | 12 |
|  | PRJNA379630 | | |
| *Plasmodium falciparum* | 1 | 5 | 5 |
|  | GSE58402 | | |
| *Streptomyces coelicolor* | 1 | 4 | 4 |
|  | GSE69350 | | |
| *Vibrio vulnificus* | 1 | 4 | 4 |
|  | GSE111991 | | |
