# Supplementary material for: riboCIRC: a comprehensive database of translatable circRNAs
Source: Genome Biol. 2021 Mar 8;22:79. doi: 10.1186/s13059-021-02300-7 (PMC7938571; doi:10.1186/s13059-021-02300-7)
Supplement: Supplementary file 2 — Additional file 2: Fig. S1. [file 13059_2021_2300_MOESM2_ESM.pdf]

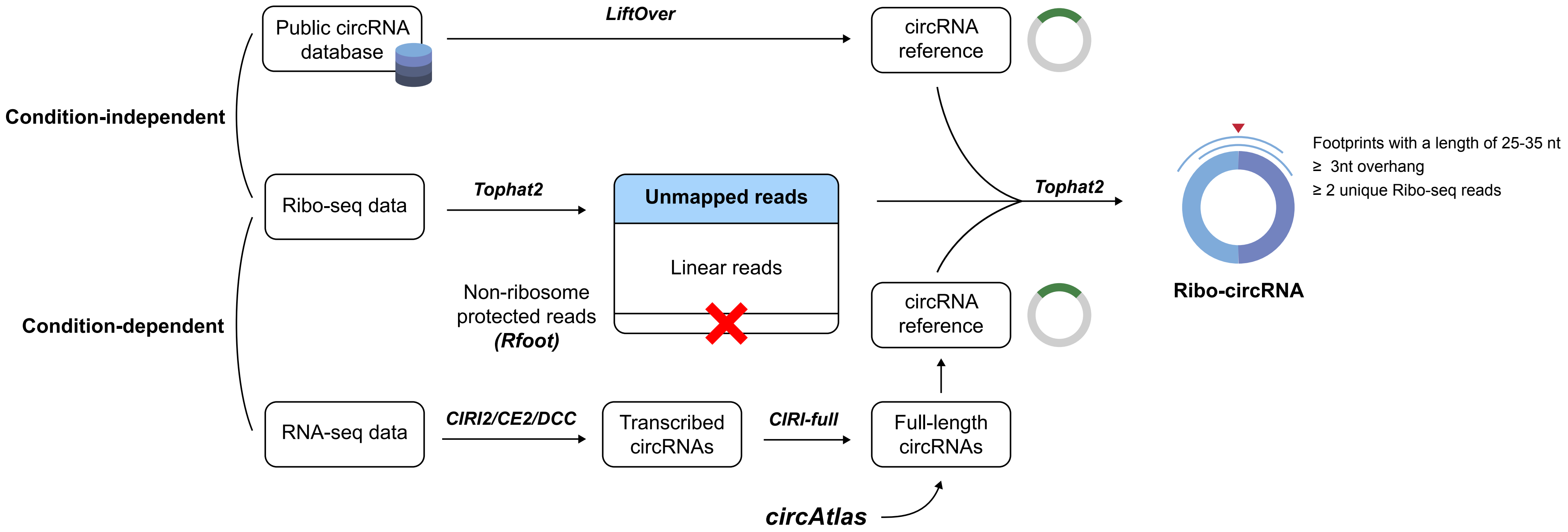

Figure S1. Flow diagram of processing pipeline for translatable circRNAs. Two different strategies were used to characterize ribosome-associated circRNAs: condition-dependent detection for Ribo-seq and perfectly matched RNA-seq datasets and condition-independent detection for previously reported circRNAs and Ribo-seq datasets.
