# Supplementary material for: riboCIRC: a comprehensive database of translatable circRNAs
Source: Genome Biol. 2021 Mar 8;22:79. doi: 10.1186/s13059-021-02300-7 (PMC7938571; doi:10.1186/s13059-021-02300-7)
Supplement: Supplementary file 3 — Additional file 3: Table S2. Summary of circRNAs reported in the public databases. [file 13059_2021_2300_MOESM3_ESM.doc]

**Supplementary Table 2. Summary of circRNAs reported in the public databases.**

| **Database** | **Species** | **Genome** | **LiftOver** | **Number of circRNAs** | **Pubmed ID** | **Note** |
| --- | --- | --- | --- | --- | --- | --- |
| circAtlas | Human | hg38 | hg38 | 580,718 | 32345360 | - |
|  | Mouse | mm10 | mm10 | 252,811 |  |  |
|  | Rat | rn6 | rn6 | 97,378 |  |  |
| circBank | Human | hg19 | hg38 | 138,414 | 31023147 | - |
| circBase | Human | hg19 | hg38 | 138,477 | 25234927 | - |
|  | Mouse | mm9 | mm10 | 16,420 |  |  |
|  | C.elegans | ce6 | ce11 | 718 |  |  |
|  | Drosophila | dm3 | dm6 | 3,406 |  |  |
| CIRCpedia | Human | hg38 | hg38 | 183,993 | 30172046 | - |
|  | Mouse | mm10 | mm10 | 55,312 |  |  |
|  | Rat | rn6 | rn6 | 10,197 |  |  |
|  | C.elegans | ce10 | ce11 | 3,840 |  |  |
|  | Drosophila | dm6 | dm6 | 8,560 |  |  |
|  | Zebrafish | danRer10 | danRer11 | 891 |  |  |
| circRNADb | Human | hg19 | hg38 | 32,472 | 27725737 | - |
| CSCD | Human/cancer | hg38 | hg38 | 507,197 | 29036403 | - |
| exoRBase | Human/blood exosomes | hg38 | hg38 | 58,330 | 30053265 | - |
| TSCD | Human | hg38 | hg38 | 128,470 | 27543790 | - |
|  | Mouse | mm10 | mm10 | 5,038 |  |  |
| Circ2Disease | Human | hg19 | hg38 | 248 | 30030469 | - |
| MiOncoCirc | - | - | - | - | 30735636 | incomplete annotation |
| Circad | - | - | - | - | 32219412 | lack of a download link |
| CircFunBase | - | - | - | - | 30715276 | lack of a download link |
| CircR2disease | - | - | - | - | 29741596 | incomplete annotation |
| CircRNADisease | - | - | - | - | 29700306 | incomplete annotation |
| LncRNADisease | - | - | - | - | 23175614 | incomplete annotation |
| CircInteractome | - | - | - | - | 26669964 | lack of a download link |
| CircNet | - | - | - | - | 26450965 | Inaccessible webpage |
