# Supplementary material for: riboCIRC: a comprehensive database of translatable circRNAs
Source: Genome Biol. 2021 Mar 8;22:79. doi: 10.1186/s13059-021-02300-7 (PMC7938571; doi:10.1186/s13059-021-02300-7)
Supplement: Supplementary file 4 — Additional file 4: Table S3. Summary of public proteomics datasets used in this study. [file 13059_2021_2300_MOESM4_ESM.doc]

**Supplementary Table S3. Summary of public proteomics datasets used in this study**

| **Species** | **PXD** | **Tissue/Cell line** | **Samples (.raw)** | **Number of cORFs with peptide hits** | **Number of ribo-circRNAs with peptide hits** |
| --- | --- | --- | --- | --- | --- |
| Human | PXD018569 | Lung cell line | 216 | 51 | 484 |
|  | PXD018570 | Lung cell line | 214 | 55 |  |
|  | PXD018571 | Lung cell line | 239 | 55 |  |
|  | PXD018572 | Lung cell line | 216 | 54 |  |
|  | PXD018573 | Lung cell line | 214 | 52 |  |
|  | PXD018574 | Lung cell line | 212 | 126 |  |
|  | PXD001406 | LCLs | 42 | 4 |  |
|  | PXD002389 | HEK293 | 100 | 10 |  |
|  | PXD002395 | 11 human cell lines | 198 | 42 |  |
|  | PXD016999 | 32 normal human tissues | 672 | 87 |  |
|  | PXD007203 | Foreskin fibroblasts | 6 | 2 |  |
|  | PXD017159 | Blood/T lymphocyte | 210 | 177 |  |
|  | PXD021391 | Multiple healthy human tissues | 723 | 168 |  |
| Mouse | PXD013502 | Brain | 151 | 20 | 184 |
|  | PXD013892 | Kidney inner medulla | 20 | 0 |  |
|  | PXD019880 | Spleen/Liver/Lung/Muscle/Kidney/Brain/Heart | 8 | 0 |  |
|  | PXD020091 | Lymphoid and myeloid populations | 192 | 37 |  |
|  | PXD023256 | Lymph node/T cell | 120 | 40 |  |
|  | PXD014512 | Liver | 40 | 26 |  |
|  | PXD000867 | Liver | 177 | 112 |  |
| Rat | PXD015427 | Spleen/Liver/Cell culture/Lung/Kidney/Testis | 1145 | 0 | 0 |
|  | PXD006349 | Brain | 20 | 0 |  |
| Drosophila | PXD007669 | S2 cell line | 48 | 0 | 0 |
|  | PXD000455 | Whole fly | 119 | 0 |  |
| C.elegans | PXD004561 | Whole body | 82 | 2 | 1 |
| Zebrafish | PXD000479 | Eye/Brain/Liver/Spleen/Intestine-pancreas/Ovary/Testes/ Muscle/Heart/Head | 64 | 0 | 0 |
| Total | 26 | - | 5,548 | 719 (unique cORFs) | 669 (unique circRNAs) |
