# Supplementary material for: riboCIRC: a comprehensive database of translatable circRNAs
Source: Genome Biol. 2021 Mar 8;22:79. doi: 10.1186/s13059-021-02300-7 (PMC7938571; doi:10.1186/s13059-021-02300-7)
Supplement: Supplementary file 5 — Additional file 5. Review history. [file 13059_2021_2300_MOESM5_ESM.docx]

Review History

**First round of review**

**Reviewer 1**

**Are you able to assess all statistics in the manuscript, including the appropriateness of statistical tests used?** Yes: I have assessed the statistics in the text. Generally, the statistical methods used were appropriate for the tests.

**Comments to author:**

This study presented a translated circRNAs database, riboCIRC. A large number of Ribo-seq and RNA-seq data were collected and used to determine translation potential of the collected circRNA candidates. The current version of riboCIRC contains a total of 2,265 computational predicted and 56 experimentally validated translated circRNAs. Some concerns are listed below.

1. Numerous currently available databases also provide translated circRNA predictions such as circAtlas, circbank, circRNADb, CSCD, CircInteractome, and TransCirc. The authors should make a comprehensive comparisons between their database and these databases. Particularly, the functions of riboCIRC and TransCirc (NAR, 2020) highly overlap with each other. Of note, TransCirc used 7 different types of data/methods to predict circRNA translation potential, whereas riboCirc only used ribo-seq data and ORF prediction. Two widely used signals, IRES and m6A, were not considered in riboCIRC. The novelty and advantages of riboCIRC should be pointed out.

2. Since the study claimed that riboCIRC included translated circRNAs from 21 species, riboCIRC can provide conserved translated circRNAs. By the way, the authors claimed that the study involved translated circRNAs across 21 species. However, only circRNAs from 6 species were found in riboCIRC.

3. Line 49, Page 4: "After excluding reads protected by nonribosomal complexes from the Ribo-seq datasets using Rfoot, all the remaining reads that were unmapped to the corresponding linear reference genome were then realigned to the pseudo circRNA reference using Tophat2." The definition of unmapped reads (Fig.S1) was unclear. Should the unmapped reads satisfy the role of 3-nt periodicity?

4. It is necessary to determine the full-length and alternative splicing of circRNAs before the prediction of circRNA ORFs. Several tools such as CIRI-AS, CIRI-full, or CircAST were developed to reconstruct full-length circRNAs and multiple circRNA isoforms.

5. Line 45, Page 5: "Briefly, the exonic sequence of each circRNA was retrieved and multiplied four times". The authors did not explain why the exonic sequence of each circRNA requires to multiple four times. Why not three times?

6. Regarding experimentally validated translated circRNAs, some PMIDs are missing at the website. At least, the origin source should be provided

**Reviewer 2**

**Are you able to assess all statistics in the manuscript, including the appropriateness of statistical tests used?** Yes: not applicable.

**Comments to author:**

The authors have compiled a comprehensive resource of translatable circRNA.

While I deem the underlying research question highly relevant and the database as essential resource, I have a few specific issues that I would like to see addressed:

1) In the abstract, you write:

The riboCIRC database is specifically designed for hosting, exploring, analyzing, and visualizing translated circRNAs.

The database provides: (1) a comprehensive repository of computationally predicted translated circRNAs based on 3,168 ribosome profiling and 1,970 RNA sequencing public datasets;

The term "translated" is simply wrong in my humble opinion.. you could possibly say, at best, that the circRNAs are ribosome associated. The criteria as outlined in the methods section are not sufficient to claim translation.

2) Later on you say: (3) a systematic de novo annotation of translated circRNA-encoded peptides, including sequence, structure, and function;

There is a complete absence of any mass spectrometry data (proteomics) whatsoever. An inclusion and analyses of mass-spectrometry evidence is mandatory.

What is also crucial in this context, is proper statistical estimation of False discovery rates for peptide identification in a database of linear and circular ORFs.

3) Wrt Bioinformatics methods: I am not happy with a step-wise mapping procedure. For example, the STAR aligner enables (on the fly) simultaneous mapping to linear and circular RNA templates.

I look forward to receiving a carefully revised version of the manuscript.

**Authors Response**

**Point-by-point responses to the reviewers’ comments:**

This study presented a translated circRNAs database, riboCIRC. A large number of Ribo-seq and RNA-seq data were collected and used to determine translation potential of the collected circRNA candidates. The current version of riboCIRC contains a total of 2,265 computational predicted and 56 experimentally validated translated circRNAs. Some concerns are listed below.

**Reply:** Thank you very much for your thoughtful comments. We gave a point-by-point reply to your comments below.

1. Numerous currently available databases also provide translated circRNA predictions such as circAtlas, circbank, circRNADb, CSCD, CircInteractome, and TransCirc. The authors should make a comprehensive comparison between their database and these databases. Particularly, the functions of riboCIRC and TransCirc (NAR, 2020) highly overlap with each other. Of note, TransCirc used 7 different types of data/methods to predict circRNA translation potential, whereas riboCirc only used ribo-seq data and ORF prediction. Two widely used signals, IRES and m6A, were not considered in riboCIRC. The novelty and advantages of riboCIRC should be pointed out.

**Reply:** Here we summarized the major differences between our and other circRNA databases:

(**a**) Different from those transcriptome data-oriented circRNA databases such as circAtlas, circbank, circRNADb, CSCD, CircInteractome, riboCIRC is a translatome data-oriented database that aims to provide direct *in vivo* translation evidence for circRNAs.

(**b**) During the review process of riboCIRC, TransCirc is published. Compared to TransCirc, riboCIRC (***i***) is a dedicated multi-species translatable circRNA database, instead TransCirc only provides information about the circRNAs from human, (***ii***) provides the largest manually curated collection of experimentally verified translated circRNAs; (***iii***) provides an intuitive view and comparison of ribosome footprints in circRNAs by JBrowse browser, (***iv***) includes a systematic *de novo* annotation of circRNA-encoded peptides, including sequence, structure, and function, and (***v***) contains an evaluation of cross-species conservation of translatable circRNAs, as added in the revision.

In addition, per the suggestion, we have annotated IRES elements and m6A sites in circRNAs in the revision. We have updated the Abstract and the manuscript accordingly.

2. Since the study claimed that riboCIRC included translated circRNAs from 21 species, riboCIRC can provide conserved translated circRNAs. By the way, the authors claimed that the study involved translated circRNAs across 21 species. However, only circRNAs from 6 species were found in riboCIRC.

**Reply:** Thank you for your valuable suggestion on providing conserved translatable circRNAs. As suggested, we have performed the cross-species conservation analysis of translatable circRNAs and added this information in the revision.

Indeed, we analyzed 3,168 public Ribo-seq and 1,970 matched RNA-seq datasets from 21 different species. Translatable circRNAs could eventually be detected in 6 out of 21 species. This is why only circRNAs from 6 species were included in our database. We have revised the manuscript to make this clear.

3. Line 49, Page 4: "After excluding reads protected by nonribosomal complexes from the Ribo-seq datasets using Rfoot, all the remaining reads that were unmapped to the corresponding linear reference genome were then realigned to the pseudo circRNA reference using Tophat2." The definition of unmapped reads (Fig.S1) was unclear. Should the unmapped reads satisfy the role of 3-nt periodicity?

**Reply:** We are sorry for this unclear description. In the revised version, we have modified the Figure S1 and rewritten these sentences to make it clear: “*After the removal of footprints from nonribosomal complexes, all the ribosome-protected footprints were then mapped with Tophat2 to the corresponding linear reference genome, and the resulting unmapped.bam file was further remapped to the pseudo circRNA reference using Tophat2, with default parameters except N, which was set to 0 (the default is 2)*”. However, given the unknown genomic positions for most of the unmapped reads, we cannot determine their 3-nt periodicity.

4. It is necessary to determine the full-length and alternative splicing of circRNAs before the prediction of circRNA ORFs. Several tools such as CIRI-AS, CIRI-full, or CircAST were developed to reconstruct full-length circRNAs and multiple circRNA isoforms.

**Reply:** We appreciate the comments. As suggested, the full-length sequence of each identified circRNA was assembled by the CIRC-full pipeline or extracted from the circAtlas database when the RNA-seq data were unavailable. In the revised version, we have added a detailed description for getting the full-length sequence of each circRNA. We also modified some corresponding contents in our revised version of database, including potential cORFs and their sequence, structure and function annotation.

5. Line 45, Page 5: "Briefly, the exonic sequence of each circRNA was retrieved and multiplied four times". The authors did not explain why the exonic sequence of each circRNA requires to multiple four times. Why not three times?

**Reply:** Thanks for pointing this out. As guided by the pioneer paper (*Mol Cell. 2017 Apr 6; 66(1): 9-21.e7*), we used the same strategy to multiply four times to detect potential rolling translated ORFs. In the revised version, this sentence was modified to make it clear and the reference was included: “*Briefly, the full-length sequence of each ribo-circRNA was retrieved and multiplied four times to allow for rolling circle translation*”.

6. Regarding experimentally validated translated circRNAs, some PMIDs are missing at the website. At least, the origin source should be provided

**Reply:** Per the suggestion, we have filled in all the missing information. In addition, we have updated the recently reported novel translated circRNAs, making the total number from 56 to 216.

**Reviewer #2:**

The authors have compiled a comprehensive resource of translatable circRNA. While I deem the underlying research question highly relevant and the database as essential resource, I have a few specific issues that I would like to see addressed:

**Reply:** We highly appreciate the reviewer’s insightful and helpful comments on our database. Thank you very much for your effort. Please find below our responses to each of your comments.

1) In the abstract, you write:

The riboCIRC database is specifically designed for hosting, exploring, analyzing, and visualizing translated circRNAs. The database provides: (1) a comprehensive repository of computationally predicted translated circRNAs based on 3,168 ribosome profiling and 1,970 RNA sequencing public datasets; The term "translated" is simply wrong in my humble opinion. you could possibly say, at best, that the circRNAs are ribosome associated. The criteria as outlined in the methods section are not sufficient to claim translation.

**Reply:** Thanks for pointing this out. In the revised version, we have toned-down this aspect and replaced “translated circRNA” with “ribosome-associated circRNAs”.

2) Later on you say: (3) a systematic de novo annotation of translated circRNA-encoded peptides, including sequence, structure, and function; There is a complete absence of any mass spectrometry data (proteomics) whatsoever. An inclusion and analyses of mass-spectrometry evidence is mandatory. What is also crucial in this context, is proper statistical estimation of False discovery rates for peptide identification in a database of linear and circular ORFs.

**Reply:** We appreciate the comments. Per the suggestion, we downloaded 24 public proteomics datasets from the PRIDE database. In the revision, we provided protein evidence for 498 unique cORF-encoded peptides from 472 circRNAs. Compared to the recently published TransCirc database, where 168 out of 328,080 circRNAs had peptide evidence, our database had a much higher ratio of circRNAs with peptide evidence. As we are still searching the proteomics datasets, the number of circRNAs with peptide evidence is expected to keep increasing. We will continue to update the database with more proteomics datasets included and eventually have an estimation of FDR for peptide identification.

Although junction-spanning peptides provide direct evidence for the translation of circRNAs, non-detection of such peptides does not rule out translation of circRNAs. The reasons are summarized as follows: (1) some junction-spanning peptides likely escape detection due to extremely low expression abundance; (2) some junction-spanning peptides are likely rapidly degraded during proofreading through nonsense-mediated decay; and (3) only a limited amount of proteomics datasets is currently used for providing proof of junction-spanning peptides. Therefore, as suggested by Reviewer 1, we have additionally provided several types of evidence to support circRNA translation, including IRES elements and m6A modification, given the ability of IRES elements and m6A modification to drive circRNA translation (*Mol Cell. 2017 Apr 6;66(1):9-21.e7;* *Cell Res. 2017 May;27(5):626-641*).

3) Wrt Bioinformatics methods: I am not happy with a step-wise mapping procedure. For example, the STAR aligner enables (on the fly) simultaneous mapping to linear and circular RNA templates.

**Reply:** Thank you very much for the suggestion. At the beginning, we also tried the simultaneous mapping strategy and expected to define ribosome-associated circRNAs using backsplice junctions direct extracted from the chimeric alignments. However, given that ribosome-protected footprints have a typical length range of 25-35 nt, tightly distributed around a peak of ~30 nt, it is very difficult to detect back-splice junction reads from Ribo-seq data because sequencing read length had a dramatic effect on the detection of these chimeric reads, with shorter reads leading to a relatively low sensitivity (*Genome Biol. 2019 Oct 21;20(1):213*). Therefore, we used a step-wise mapping in our analysis, as done in the previous papers (*Cell. 2019 Jun 27;178(1):242-260.e29;* *Mol Cell. 2017 Apr 6;66(1):9-21.e7*). We will update our identification strategy in future as more robust tools are developed.

**Second round of review**

**Reviewer 1**

The authors have addressed most of concerns instead of a minor point. Since the full lengths of circRNAs were generated by the CIRI-full pipeline or extracted from the circAltas database, it would be better to present the full lengths and the corresponding sequences of circRNAs in riboCIRC.

**Reviewer 2**

The authors have compiled a comprehensive resource of translatable circRNA. While I deem the underlying research question highly relevant and the database as essential resource, I have a few specific issues that I would like to see addressed:

Reply: We highly appreciate the reviewer's insightful and helpful comments on our database. Thank you very much for your effort. Please find below our responses to each of your comments.

1) In the abstract, you write:

The riboCIRC database is specifically designed for hosting, exploring, analyzing, and visualizing translated circRNAs. The database provides: (1) a comprehensive repository of computationally predicted translated circRNAs based on 3,168 ribosome profiling and 1,970 RNA sequencing public datasets; The term "translated" is simply wrong in my humble opinion. you could possibly say, at best, that the circRNAs are ribosome associated. The criteria as outlined in the methods section are not sufficient to claim translation.

Reply: Thanks for pointing this out. In the revised version, we have toned-down this aspect and replaced "translated circRNA" with "ribosome-associated circRNAs".

R#2 Comment: Perfect. This addresses my concerns.

2) Later on you say: (3) a systematic de novo annotation of translated circRNA-encoded peptides, including sequence, structure, and function; There is a complete absence of any mass spectrometry data (proteomics) whatsoever. An inclusion and analyses of mass-spectrometry evidence is mandatory. What is also crucial in this context, is proper statistical estimation of False discovery rates for peptide identification in a database of linear and circular ORFs.

Reply: We appreciate the comments. Per the suggestion, we downloaded 24 public proteomics datasets from the PRIDE database. In the revision, we provided protein evidence for 498 unique cORF-encoded peptides from 472 circRNAs. Compared to the recently published TransCirc database, where 168 out of 328,080 circRNAs had peptide evidence, our database had a much higher ratio of circRNAs with peptide evidence. As we are still searching the proteomics datasets, the number of circRNAs with peptide evidence is expected to keep increasing. We will continue to update the database with more proteomics datasets included and eventually have an estimation of FDR for peptide identification.

Although junction-spanning peptides provide direct evidence for the translation of circRNAs, non-detection of such peptides does not rule out translation of circRNAs. The reasons are summarized as follows: (1) some junction-spanning peptides likely escape detection due to extremely low expression abundance; (2) some junction-spanning peptides are likely rapidly degraded during proofreading through nonsense-mediated decay; and (3) only a limited amount of proteomics datasets is currently used for providing proof of junction-spanning peptides. Therefore, as suggested by Reviewer 1, we have additionally provided several types of evidence to support circRNA translation, including IRES elements and m6A modification, given the ability of IRES elements and m6A modification to drive circRNA translation (Mol Cell. 2017 Apr 6;66(1):9-21.e7; Cell Res. 2017 May;27(5):626-641).

R#2 Comment: Makes sense. Please add information on the respective database size for each species. It is unclear how many ORFs were tested in the MQ search. Would be useful to provide these databases as download (FASTA AA) too.

3) Wrt Bioinformatics methods: I am not happy with a step-wise mapping procedure. For example, the STAR aligner enables (on the fly) simultaneous mapping to linear and circular RNA templates.

Reply: Thank you very much for the suggestion. At the beginning, we also tried the simultaneous mapping strategy and expected to define ribosome-associated circRNAs using backsplice junctions direct extracted from the chimeric alignments. However, given that ribosome-protected footprints have a typical length range of 25-35 nt, tightly distributed around a peak of ~30 nt, it is very difficult to detect back-splice junction reads from Ribo-seq data because sequencing read length had a dramatic effect on the detection of these chimeric reads, with shorter reads leading to a relatively low sensitivity (Genome Biol. 2019 Oct 21;20(1):213). Therefore, we used a step-wise mapping in our analysis, as done in the previous papers (Cell. 2019 Jun 27;178(1):242-260.e29; Mol Cell. 2017 Apr 6;66(1):9-21.e7). We will update our identification strategy in future as more robust tools are developed.

R#2 Comment: Fine for now. Looking fwd to your updates.

**Authors Response**

**Point-by-point responses to the reviewers’ comments:**

Reviewer #1:

The authors have addressed most of concerns instead of a minor point. Since the full

lengths of circRNAs were generated by the CIRI-full pipeline or extracted from the

circAltas database, it would be better to present the full lengths and the corresponding

sequences of circRNAs in riboCIRC.

Reply: Thanks for the suggestion. We have done it accordingly. Please click the

riboCIRC ID on the Ribo-circRNA page to view the information, which is also available

on the download page.

Reviewer #2:

Comment on Question 2: Makes sense. Please add information on the respective

database size for each species. It is unclear how many ORFs were tested in the MQ

search. Would be useful to provide these databases as download (FASTA AA) too.

Reply: Thanks for the suggestion. As suggested, we have added information on the

respective database size for each species in the revised text and also provided these

customized protein sequence databases on the download page.
